# Ferroelectric Single‐Molecule Magnet with Toroidal Magnetic Moments

**DOI:** 10.1002/advs.202202979

**Published:** 2022-07-20

**Authors:** Yu‐Xia Wang, Yinina Ma, Jie‐Su Wang, Yue Yang, Yun‐Nan Guo, Yi‐Quan Zhang, Kui‐Juan Jin, Young Sun, Peng Cheng

**Affiliations:** ^1^ Key Laboratory of Advanced Energy Materials Chemistry (MOE) Haihe Laboratory of Sustainable Chemical Transformations (Tianjin) Renewable Energy Conversion and Storage Center College of Chemistry Nankai University Tianjin 300071 P. R. China; ^2^ Institute of Physics Chinese Academy of Sciences Beijing 100190 P. R. China; ^3^ Division of Quantum States of Matter Beijing Academy of Quantum Information Sciences Beijing 100193 China; ^4^ Department of Chemistry Zhejiang Sci‐Tech University Hangzhou 310018 P. R. China; ^5^ Jiangsu Key Lab for NSLSCS School of Physical Science and Technology Nanjing Normal University Nanjing 210023 P. R. China; ^6^ Center of Quantum Materials and Devices and Department of Applied Physics Chongqing University Chongqing 401331 P. R. China

**Keywords:** multiferroic materials, second‐harmonic generation, single‐molecule magnets, toroidal magnetic moments

## Abstract

Materials that coexist magnetic and electric properties on the molecular scale in single‐molecule magnets (SMMs) with peculiar quantum behaviors have promise in molecular electronics and spintronics. Nevertheless, such molecular materials are limited in potentials because their magnetic signal cannot be transformed into an electrical signal through magnetoresistance or Hall effects for their high insulativity. The discovery of an entirely new material, ferroelectric SMMs (FE SMMs) is reported. This FE SMM also shows single‐molecule magnetic behaviors, toroidal magnetic moments, and room‐temperature ferroelectricity. The toroidal moment is formed by a vortex distribution of magnetic dipoles in triangular Dy_3_ clusters. The analysis of ac magnetic susceptibility reveals the coexistence of three distinct magnetic relaxation processes at low temperatures. The ferroelectricity is introduced by incorporating polar alcohol molecules in the structure, which is confirmed by the X‐ray diffraction and optical second harmonic generation (SHG) measurements. Moreover, the dielectric measurements reveal a ferroelectric‐to‐ferroelectric phase transition around 150 K due to the symmetry change from *P*c to *P*na2_1_. The coexistence of toroidal moment and ferroelectricity along with quantum magnetism in the rare‐earth single‐molecule magnets yields a unique class of multiferroics.

## Graphical abstract

Multiferroic materials are exploded in the molecular magnetic materials in this work. A ferroelectric single‐molecule magnet is characterized by three unprecedented slow magnetic relaxations and ferroelectricity.

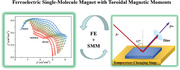

## 1 Introduction

Single‐molecule magnets (SMMs) are discrete molecular species with quantum tunneling behaviors of magnetism.^[^ ^1^ ^]^ Many SMMs with different structures and magnetic ions have been designed and synthesized in the past two decades.^[^ ^2^, ^3^, ^4^, ^5^ ^]^ Recently, SMMs with fantastic toroidal moments have attracted much attention owing to their special spin arrangement and abundant quantum magnetism,^[^ ^6^ ^]^ where the toroidal moment is characterized by a vortex distribution of magnetic dipoles.^[^ ^7^ ^]^ The toroidal moment is regarded as an electromagnetic moment in addition to traditional electric polarization and magnetization,^[^ ^8^ ^]^ and the concept of multiferroics has been extended to include the ferro‐toroidal order.^[^ ^9^ ^]^ A lot of intriguing properties based on toroidal moments have been observed in recent years.^[^ ^10^, ^11^, ^12^, ^13^, ^14^ ^]^

Although the magnetic properties of SMMs have been extensively studied, their electric properties have been less concerned because they are generally insulating dielectrics without magnetoresistance or Hall effects. This feature makes SMMs unfavorable for applications in electronic and spintronic devices.^[^ ^15^, ^16^ ^]^ However, when ferroelectricity is introduced into SMMs, their functionality would be greatly expanded in terms of multiferroicity and magnetoelectric effects. In fact, there has been growing interest in achieving ferroelectricity in organic molecular materials and metal‐organic frameworks beyond traditional ferroelectric oxides.^[^ ^17^, ^18^, ^19^ ^]^ Nevertheless, ferroelectricity in SMMs has not been convincingly achieved. In this letter, we report the discovery of ferroelectricity above room temperature in a peculiar SMM with toroidal magnetic moments.

In this work, we constructed and characterized the solid evidences of SMM and ferroelectricity in a ferroelectric SMM,^[^ ^20^ ^]^ [Dy_3_(HL)(H_2_L)(NO_3_)_4_]·C_2_H_5_OH (H_4_L = *N*,*N*,*N*’,*N*’‐tetrakis(2‐hydroxyethyl)‐ethylene‐diamine), which posesses toroidal moment formed by a vortex distribution of magnetic dipoles in triangular Dy_3_ clusters. Interestingly, we find three‐step magnetic relaxation processes coexisting in the same temperature range. A modified Debye model with three relaxation times was introduced to study the multiple dynamic mechanisms as well. The ferroelectricity with the Curie temperatures *T*_C_ ≈ 470 K was evidenced by the SHG signal that is only allowed in non‐centrosymmetric structures. Moreover, the dielectric anomaly around 160–170 K suggests a ferroelectric‐to‐ferroelectric phase transition originated from the structural transformation from *P*c to *P*na2_1_.

## 2 Results and Discussion

**Figure 1:**
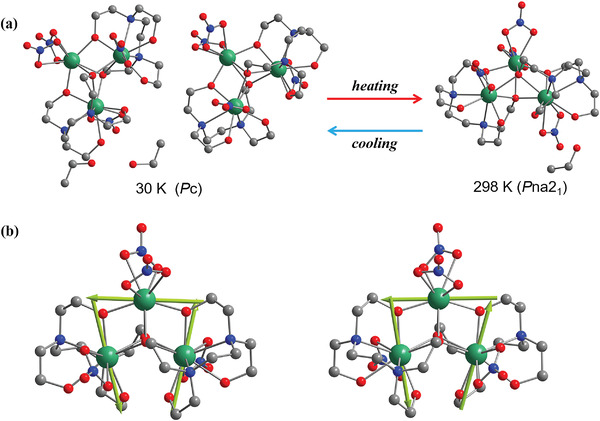
Crystalline structure and toroidal magnetic moments. a) Crystal structure and space group at 298 and 30 K. b) Toroidal magnetic moments in two configurations of Dy_3_ clusters at low temperatures. The arrows represent the calculated orientations of the local magnetic easy axes on Dy^3+^ ions. Color scheme: green Dy, red O, grey C, and blue N. The hydrogen atoms and the isopropanol molecules are omitted for clarity.

X‐ray diffraction studies reveal that the SMM crystallizes in the polar space group *P*na2_1_ at 298 K (**Figure** **1**a). There are three crystallography‐independent Dy^3+^ ions and one alcohol molecule in the polar *P*na2_1_ space group. One eight‐coordinated Dy^3+^ ion (Dy1) is coordinated by eight oxygen atoms (DyO_8_) from two nitrates and two ligands, while the other two nine‐coordinated Dy^3+^ ions (Dy2 and Dy3) are surrounded by two nitrogen atoms and seven oxygen atoms (DyN_2_O_7_) provided by one nitrate and two ligands. At 30 K, the space group changes to another polar space group *P*c. The main difference between the crystal structures of *P*na2_1_ and *P*c is the *Z*‐value and the ordering of solvent alcohol molecule. As a result, there are two types of molecular structure in the polar *P*c space group, as shown in Figure 1b. Four different orientation triangular molecules and four ethanol molecules surround the 2_1_ screw axis in the *P*na2_1_ phase but two different orientation triangular molecules and two ethanol molecules in the *P*c phase which induced different molecular packing. The local coordination symmetry of each Dy^3+^ center was analyzed by continuous shape measure ^[^ ^21^ ^]^ calculations on the DyO_8_ and DyN_2_O_7_ sites. At both 298 and 30 K, the calculations reveal that Dy1 is situated in a slightly distorted *D*_4d_ geometry while Dy2 and Dy3 are situated within a *C*_4v_ coordination environment with different deviation values.

Based on the determined geometries of the Dy_3_ complex at 30 K, Complete‐active‐space self‐consistent field (CASSCF) calculations on individual Dy^3+^ fragments were carried out. The energy levels, ***g*** tensors, and the predominant *m_J_* values of the lowest eight Kramers doublets (KDs) of individual Dy^3+^ fragments were obtained. POLY_ANISO program^[^ ^22^, ^23^, ^24^, ^25^ ^]^ was used to fit the Dy^3+^‐Dy^3+^ exchange interactions and the intermolecular interactions through comparison of the computed and measured magnetic susceptibilities. The computational details are described in the Supporting Information. The results suggest that the Dy^3+^‐Dy^3+^ interactions in the Dy_3_ clusters are antiferromagnetic. The magnetic axes on Dy^3+^ ions in the two molecular structures are indicated in Figure 1b. The included angles between magnetic easy axes of Dy^3+^ ions are all larger than 97^o^. Therefore, the local magnetization vectors of Dy^3+^ ions can be approximately regarded as a triangle. Subsequently, a toroidal moment is formed based on the Dy_3_ magnetic triangles.

**Figure 2:**
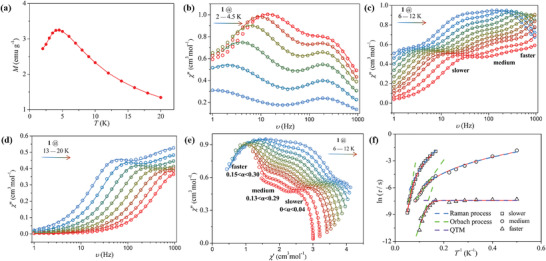
Magnetic relaxation analyses. a) Temperature dependence of magnetization measured in 1000 Oe. b–d) Imaginary component of ac magnetic susceptibility as a function of frequency in different temperature ranges. e) The Cole‐Cole plots at 6–12 K show three distinct magnetic relaxation processes, named faster, medium, and slower, respectively. f) The relaxation time ln(*τ*) as a function of the reciprocal of temperature. The different relaxation processes are marked in different symbols. The solid lines are the best fitting results using the sum of three modified Debye functions.

A vanishing small magnetic susceptibility at low temperatures would be expected for the state with toroidal magnetic moments, as reported in previous studies.^[^ ^20^ ^]^ **Figure** **2**a shows the temperature dependence of dc magnetization of the Dy_3_ complex. The magnetization exhibits a maximum of around 4.5 K and decreases toward zero at low temperatures. This feature is consistent with the anticipated behavior of a SMM with toroidal magnetic moments.

The ac magnetic susceptibility of the Dy_3_ complex exhibits a strongly frequency‐dependent behavior in the temperature range from 2 to 20 K (see Figure S1, Supporting Information), which is another characteristic of SMMs. Especially, the imaginary component (*χ*“) shows an unusual feature of multiple peaks, indicating multiple magnetic relaxation processes. In the low‐temperature region (2–4.5 K), *χ*” shows two separate peaks, corresponding to a slow (low frequency) and fast (high frequency) relaxation process, respectively, as shown in Figure 2b. While the slow relaxation shifts to higher frequencies with increasing temperature, the fast relaxation process is almost temperature‐independent, which suggests that the fast relaxation is related to quantum tunneling of magnetization (QTM).^[^ ^26^ ^]^ In the intermediate temperature region (6–12 K), three distinguishable relaxation processes, named as the faster, medium, and slower processes, coexist (Figure 2c). Both the slower and medium relaxations shift to higher frequencies as temperature increases. In the high‐temperature region, the medium relaxation process gradually shifts to high frequencies and becomes undetectable while the slow relaxation process remains (Figure 2d). The strong shift with the temperature of the slower and medium relaxation processes indicates that they are related to thermally activated processes.

Interestingly, the experimental data between 6 and 12 K can be nicely simulated and depicted as the Cole–Cole plots^[^ ^27^ ^]^ which provide a more indicative view of the evolution and coexistence of multiple relaxation processes, as shown in Figure 2e. The relaxation time (*τ*) for each process can be extracted by fitting the *χ*"(*ν*) curves to three relaxation processes using the sum of three modified Debye functions,^[^ ^28^ ^]^

(1) $$\begin{array}{c} \chi_{\mathrm{ac}}\left(\omega \right)=\chi_{S}+\sum_{k}\frac{\Delta \chi_{k}}{1+{\left(i\omega \tau \right)}^{\left(1-\alpha_{{}_{k}}\right)}}\\ \chi_{S}=\sum_{k}\chi_{S,k};\Delta \chi_{k}=\chi_{T,k}-\chi_{S,k} \end{array}$$

where *χ*_S_ = *χ*_S1_ + *χ*_S2_ + *χ*_S3_ represents the sum of the adiabatic susceptibilities of the three and two relaxing species; Δ*χ*_*κ*_ is the difference between the adiabatic susceptibility (*χ*_S,*κ*_) and the isothermal susceptibility (*χ*_T,*κ*_) of each magnetic phase; *ω* = 2*πν* and the parameter *α* is the quantifying the width of the *τ* distribution. The small *α* values (*α* < 0.30) indicate that each relaxation phase has a very narrow distribution of relaxation times. And the remainder two temperature ranges of 2 to 4.5 K and 13 to 20 K are fitted by the sum of two modified Debye functions with *χ*_S_ = *χ*_S1_ + *χ*_S2_.

The correlation between the relaxation time (*τ)* and temperature (*T*) can be obtained from a plot of ln(*τ*) versus ln(*T*) to give *n* values for the three relaxation processes (*n* is the parameter that represents the relation between the relaxation time and temperature in the equation *τ* = *T* ^–n^). These results indicate that the medium and slow processes are dominated by the Raman process and Orbach relaxation pathways, while QTM dictates the faster process (Figure 2f and Figure S2, Supporting Information).

**Figure 3:**
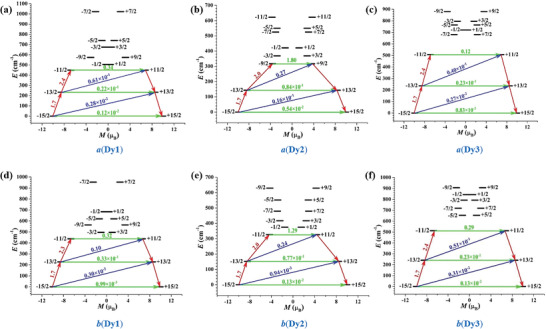
Magnetization blocking barriers of individual Dy^3+^ fragments. The thick black lines represent the KDs as a function of their magnetic moment along the magnetic axis. The green lines correspond to diagonal quantum tunneling of magnetization (QTM); the blue line represents off‐diagonal relaxation process. The numbers at each arrow stand for the mean absolute value of the corresponding matrix element of transition magnetic moment.

The corresponding magnetization blocking barriers of individual Dy^3+^ fragments are shown in **Figure** **3**, where the transversal magnetic moments in the ground KDs of individual Dy^3+^ fragments are all smaller than 10^–2^ *µ*_B_, and thus the quantum tunneling of magnetization (QTM) in their ground KDs could be suppressed at low temperature. For individual Dy^3+^ fragments of ***a*** and ***b***, the transversal magnetic moments in their first excited KDs are all smaller than 10^–1^ *µ*_B_, and thus the relaxation can probably proceed through higher excited KDs. For individual Dy^3+^ fragments of ***a*** and ***b***, the transversal magnetic moments in the second excited KDs are all larger than 10^–1^ *µ*_B_, therefore, allowing a fast QTM in their second excited KDs. Hence, the calculated energy barriers of individual Dy^3+^ fragments of them according to the scheme of Figure 3 are 450.0, 316.8, 507.0, 437.1, 326.2, and 512.2 cm^–1^, respectively. Due to the unfavorable effects of anharmonic phonons, Raman magnetic relaxation, QTM, etc on the energy barrier, the experimental effective *U*_eff_ are much smaller than the calculated energy gaps between the lowest three KDs of individual Dy^3+^ fragments.

The presence of multiple relaxation channels in SMMs is not unusual, but they usually play a dominating role in different temperature regimes. The coexistence of three relaxation processes at the same temperature has never been observed. Chibotaru et al. concluded that reorientation of the toroidal magnetization requires consecutive transitions through three exchanging excited KDs of Dy3 with the nonmagnetic ground state, whose excitation energies represent the barrier of blockage of this magnetization.^[^ ^23^ ^]^ Three exchange excited doublets for each compound are close to each other in Table S7, Supporting Information. Thus, we deduced the coexistence of three relaxation processes at the same temperature may arise from the three near‐degenerate exchange excited KDs.

**Figure 4:**
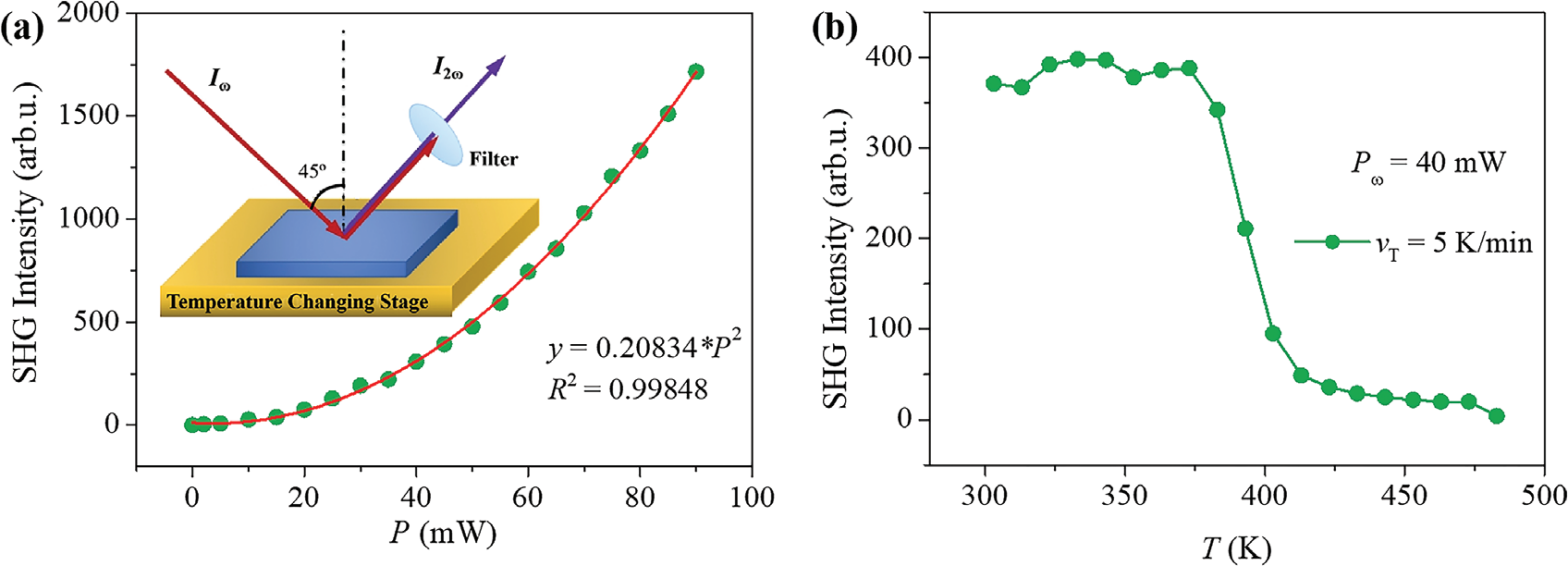
Second nonlinear response. a) Quadratic dependence of the SHG signal as a function of the laser power. As the incident light power increases, the SHG signal increases following the fitting curve *y* = A*x^2^ with a high *R* ^2^ (Adjusted R Square) value, confirming the second nonlinear response of the nanocrystals. Inset is the Schematic diagram of SHG measurement. The red line is the fitting curve to quadratic dependence. The inset shows the scheme of the SHG measurement. b) SHG intensity as a function of temperature. A ferroelectric phase transition is evidenced by a rapid increase of the intensity around 470 K.

The polar space groups at both high and low temperatures imply that this toroidal SMM could be ferroelectric. We employed the SHG technique to testify intrinsic ferroelectricity in the sample (**Figure** **4**).^[^ ^29^, ^30^, ^31^ ^]^ The relationship between the intensity of the SHG signal from a noncentrosymmetric crystal and its nonlinear polarization is *I*_(2ɷ)_/|*P*_(2ɷ)_|^2^. As shown in Figure 4a, the intensity of the reflection SHG signal of the sample presents a nonlinear variation with incident fundamental optical power, which can be well defined by the quadratic equation, confirming the second nonlinear response of the nanocrystals. In addition, the SHG signals obtained at high temperatures are almost zero. In contrast, a nonzero SHG signal is visible below ≈470 K, which corroborates the emergence of electric polarization and confirms intrinsic ferroelectricity with a Curie temperature above room temperature.

**Figure 5:**
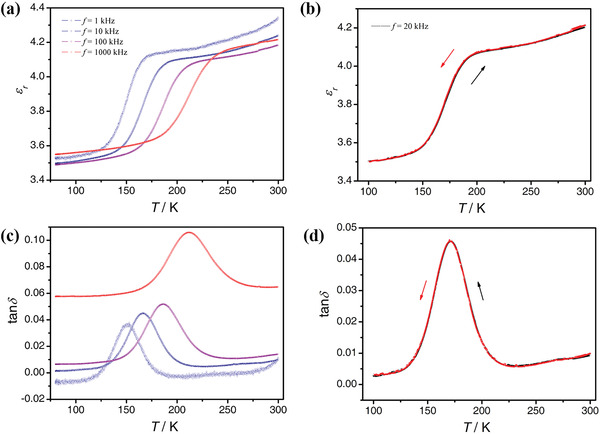
Temperature dependence of a, b) the dielectric permittivity and c,d) loss tangent of the SMM. The dielectric anomaly indicates a ferroelectric‐to‐ferroelectric phase transition around 150 K at 1 Hz.

**Figure** **5** shows the dielectric permittivity (*ε*_r_) and loss (tan*δ*) as a function of temperature between 80 and 300 K. The sudden rise of *ε*_r_ as well as the clear peak in tan*δ* indicate that there is another phase transition around 150 K. Moreover, the SHG intensity abruptly decreases to a minimum but nonzero value of around 150 K (see Figure S6, Supporting Information). The sharp decline in the SHG signal is likely due to the space group change from *P*na2_1_ to *P*c. Since both space groups are polar, this transition around 150 K corresponds to a ferroelectric–ferroelectric phase transition.

## 3 Conclusion

In summary, our study verifies a unique Dy_3_ SMM with toroidal magnetic moments and ferroelectricity, where the local magnetization vectors of Dy^3+^ ions form a triangle and the ferroelectricity is introduced by polar alcohol molecules. Interestingly, three distinct magnetic relaxation processes, corresponding to the Raman, Orbach, and QTM relaxation pathways, respectively, can coexist in the same temperature range. Hence, a modified Debye model with three relaxation times was introduced to study the multiple dynamic mechanisms. The ferroelectricity with the Curie temperatures *T*_C_ ≈ 470 K was evidenced by the SHG signal that is only allowed in noncentrosymmetric structures. Moreover, the dielectric anomaly around 150 K (1 Hz) suggests a ferroelectric‐to‐ferroelectric phase transition originated from the symmetry change from *P*c to *P*na2_1_. A significantly enhanced ME coupling would be expected in polar SMMs. This could pave the way for the design of molecular multiferroics and magnetoelectric materials using ferroelectric SMMs in the near future. The successful incorporation of ferroelectricity into SMMs not only broadens the family of multiferroics but also opens more opportunities for the electrical control of quantum magnetism of SMMs.

## 4 Experimental Section

The SMM in this study was a trinuclear (Dy_3_) complex with a formula of [Dy_3_(HL)(H_2_L)(NO_3_)_4_]·C_2_H_5_OH. The samples were synthesized by a solution method.^[^ ^20^ ^]^ Dy(NO_3_)_3_·6H_2_O (0.5 mmol, 228.3 mg) was slowly added to a 20 mL isopropanol solution containing H_4_L (1.0 mmol, 273 mg) and LiOH (0.4 mmol, 16.8 mg). The mixture was moved to a vial after 2 h of stirring. Colorless crystals were obtained under 100 ˚C solvothermal condition three days later in a 46% yield (based on Dy). Single‐crystal X‐ray diffraction was performed on an Agilent SuperNova diffractometer (298 K) and a Bruker D8 Venture diffractometer (30 K) equipped with graphite monochromated Mo‐K*α* radiation (*λ* = 0.71073 Å). The structures were solved by direct methods and refined by the full‐matrix least‐squares method on F^2^ with anisotropic thermal parameters for all nonhydrogen atoms using the SHELXL program. Hydrogen atoms were located geometrically and refined isotropically (see Table S1, Supporting Information). Powder X‐ray diffraction patterns were measured on a Rigaku Ultima IV diffractometer using Cu‐K*α* radiation (shown in Figure S7, Supporting Information). Magnetic properties were measured on powder samples using a Quantum Design superconducting quantum interference device magnetometer. The dielectric permittivity was measured on a palate made of pressed powder samples with an Aglient 4980A LCR meter in a cryogen‐free superconducting magnet system (Oxford Instruments, Teslatron PT). The optical second harmonic generation (SHG) measurements were performed by using a mode‐lock femtosecond (fs) Ti:sapphire oscillator (Tsunami 3941‐X1BB, Spectra‐Physics), which generates the incident fundamental laser with a central wavelength at 800 nm, a pulse duration of ≈100 fs, and a repetition rate of 82 MHz. The energy of the incident light was attenuated to 40 mW before being focused. A 400 nm filter was used in the reflective light path to ensure that only SH photons arrived at the photomultiplier tube. A single‐photon counting technique was conducted to count the SH photons, indicating the intensity of SHG signals generated from the samples. CASSCF calculations on individual Dy^3+^ fragments of trinuclear complex based on X‐ray determined geometries at 30 K have been carried out with MOLCAS 8.4 and SINGLE_ANISO programs.

## Conflict of Interest

The authors declare no conflict of interest.

## Supporting information

Supporting Information

## Data Availability

The data that support the findings of this study are available from the corresponding author upon reasonable request.

## References

[1] W. Wernsdorfer , R. Sessoli , Science 1994, 284, 133. 10.1126/science.284.5411.133 10102810

[2] D. N. Woodruff , R. E. P. Winpeeny , R. A. Layfield , Chem. Rev. 2013, 113, 5110. 23550940 10.1021/cr400018q

[3] S. T. Liddle , J. V. Slageren , Chem. Soc. Rev. 2015, 44, 6655. 26158197 10.1039/c5cs00222b

[4] Y.‐X. Wang , Y. Ma , Y. Chai , W. Shi , Y. Sun , P. Cheng , J. Am. Chem. Soc. 2018, 140, 7795. 29893555 10.1021/jacs.8b04818

[5] F. Paschke , T. Birk , V. Enenkel , F. Liu , V. Romankov , J. Dreiser , A. A. Popov , M. Fonin , Adv. Mater. 2021, 33, 2102844. 10.1002/adma.202102844 PMC11468252 34396601

[6] L. Ungur , S.‐Y. Lin , J. Tang , L. F. Chibotaru , Chem. Soc. Rev. 2014, 43, 6894. 24975197 10.1039/c4cs00095a

[7] J. Tang , I. Hewitt , N. T. Madhu , G. Chastanet , W. Wernsdorfer , C. E. Anson , C. Benelli , R. Sessoli , A. K. Powell , Angew. Chem., Int. Ed. 2006, 45, 1729. 10.1002/anie.200503564 16496432

[8] B. B. V. Aken , J.‐P. Rivera , H. Schmid , M. Fiebig , Nature 2007, 449, 702. 17928856 10.1038/nature06139

[9] W. Jin , E. Drueke , S. Li , A. Admasu , R. Owen , M. Day , K. Sun , S.‐W. Cheong , L. Zhao , Nat. Phys. 2020, 16, 42.

[10] L. Ungur , S. K. Langley , T. N. Hooper , B. Moubaraki , E. K. Brechin , K. S. Murray , L. F. Chibotaru , J. Am. Chem. Soc. 2020, 134, 18554. 10.1021/ja309211d 23110698

[11] S. Xue , X.‐H. Chen , L. Zhao , Y.‐N. Guo , J. Tang , Inorg. Chem. 2012, 51, 13264. 23215437 10.1021/ic301785v

[12] S.‐Y. Lin , W. Wernsdorfer , L. Ungur , A. K. Powell , Y.‐N. Guo , J. Tang , L. Zhao , L. F. Chibotaru , Angew. Chem., Int. Ed. 2012, 51, 12767. 10.1002/anie.201206602 23143895

[13] J. Luzon , K. Bernot , I. J. Hewitt , C. E. Anson , A. K. Powell , R. Sessoli , Phys. Rev. Lett. 2008, 100, 247205. 18643625 10.1103/PhysRevLett.100.247205

[14] [14] X.‐L. Liu , D. Li , H.‐X. Zhao , X.‐W. Dong , L.‐S. Long , L.‐S. Zheng , Adv. Mater. 2021, 33, 2004542. 10.1002/adma.202004542 33829543

[15] L. Bogani , W. Wernsdorfer , Nat. Mater. 2008, 7, 179. 18297126 10.1038/nmat2133

[16] M. Mannini , F. Pineider , C. Danieli , F. Totti , L. Sorace , P.h. Sainctavit , M.‐A. Arrio , E. Otero , L. Joly , J. C. Cezar , A. Cornia , R. Sessoli , Nature 2010, 468, 417. 20981008 10.1038/nature09478

[17] M. Fiebig , T. Lottermoser , D. Meier , M. Trassin , Nat. Rev. Mater. 2006, 1, 16046.

[18] L. C. Gómez‐Aguirre , B. Pato‐Doldán , J. Mira , S. Castro‐García , M. A. Señarís‐Rodríguez , M. Sanchez‐Andújar , J. Singleton , V. S. Zapf , J. Am. Chem. Soc. 2016, 138, 1122. 26717023 10.1021/jacs.5b11688

[19] Y. Tian , W. Wang , Y. Chai , J. Cong , S. Shen , L. Yan , S. Wang , X. Han , Y. Sun , Phys. Rev. Lett. 2014, 112, 017202. 24483924 10.1103/PhysRevLett.112.017202

[20] Y.‐X. Wang , W. Shi , H. Li , Y. Song , L. Fang , Y. Lan , A. K. Powell , W. Wernsdorfer , L. Ungur , L. F. Chibotaru , M. Shen , P. Cheng , Chem. Sci. 2012, 3, 3366.

[21] S. Alvarez , P. Alemany , D. Casanova , J. Cirera , M. Llunell , D. Avnir , Coord. Chem. Rev. 2005, 249, 1693.

[22] F. Aquilante , J. Autschbach , R. K. Carlson , L. F. Chibotaru , M. G. Delcey , L. De Vico , I. F. Galván , N. Ferré , L. M. Frutos , L. Gagliardi , M. Garavelli , A. Giussani , C. E. Hoyer , G. L. Manni , H. Lischka , D. Ma , P. Å. Malmqvist , T. Müller , A. Nenov , M. Olivucci , T. B. Pedersen , D. Peng , F. Plasser , B. Pritchard , M. Reiher , I. Rivalta , I. Schapiro , J. Segarra‐Martí , M. Stenrup , D. G. Truhlar , et al., J. Comput. Chem. 2016, 37, 506. 26561362 10.1002/jcc.24221

[23] L. F. Chibotaru , L. Ungur , A. Soncini , Angew. Chem., Int. Ed. 2008, 47, 4126. 10.1002/anie.200800283 18428177

[24] L. Ungur , W. Van den Heuvel , L. F. Chibotaru , New J. Chem. 2009, 33, 1224.

[25] L. F. Chibotaru , L. Ungur , C. Aronica , H. Elmoll , G. Pilet , D. Luneau , J. Am. Chem. Soc. 2008, 130, 12445. 18717564 10.1021/ja8029416

[26] M. A. Novak , W. S. D. Folly , J. P. Sinnecker , S. Soriano , J. Magn. Magn. Mater. 2005, 294, 133.

[27] K. S. Cole , R. H. Cole , J. Chem. Phys. 1941, 9, 341.

[28] Y.‐N. Guo , G.‐F. Xu , W. Wernsdorfer , L. Ungur , Y. Guo , J. Tang , H.‐J. Zhang , L. F. Chibotaru , A. K. Powell , J. Am. Chem. Soc. 2011, 133, 11948. 21744848 10.1021/ja205035g

[29] B. Zalar , V. V. Laguta , R. Blinc , Phys. Rev. Lett. 2003, 90, 037601. 12570522 10.1103/PhysRevLett.90.037601

[30] X. Feng , Y. Lun , X. Jiang , J. Qiu , H. Yu , S. Zhou , Adv. Mater 2021, 33, 2006482. 10.1002/adma.202006482 33742505

[31] A. M. Pugachev , V. I. Kovalevskii , N. V. Surovtsev , S. Kojima , S. A. Prosandeev , I. P. Raevski , S. I. Raevskaya , Phys. Rev. Lett. 2012, 108, 247601. 23004330 10.1103/PhysRevLett.108.247601

