# Supplementary material for: Ferroelectric Single‐Molecule Magnet with Toroidal Magnetic Moments
Source: Adv Sci (Weinh). 2022 Jul 20;9(26):2202979. doi: 10.1002/advs.202202979 (PMC9475528; doi:10.1002/advs.202202979)
Supplement: Supplementary file 1 — Supporting Information [file ADVS-9-2202979-s001.pdf]

ADVANCED SCIENCE Open Access

Supporting Information

for *Adv. Sci.*, DOI 10.1002/advs.202202979

Ferroelectric Single-Molecule Magnet with Toroidal Magnetic Moments

Yu-Xia Wang, Yinina Ma, Jie-Su Wang, Yue Yang, Yun-Nan Guo, Yi-Quan Zhang, Kui-Juan Jin, Young Sun* and Peng Cheng**

Supporting Information

Ferroelectric Single-Molecule Magnet with Toroidal Magnetic Moments

Yu-Xia Wang, Yinina Ma, Jie-Su Wang, Yue Yang, Yun-Nan Guo, Yi-Quan Zhang, Kui-Juan Jin, Young Sun*, and Peng Cheng**

1. Magnetic characterization

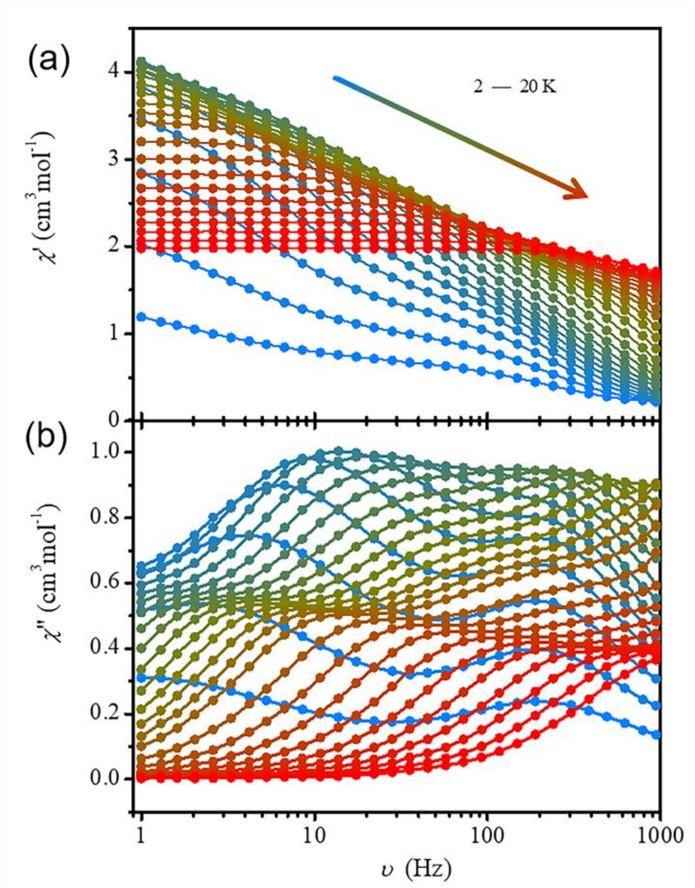

Figure S1. The real (χ') and imaginary (χ'') components of ac molar magnetic susceptibility as a function of frequency under zero dc field in the temperature range from 2 to 20 K. The solid lines are a guide to eyes.

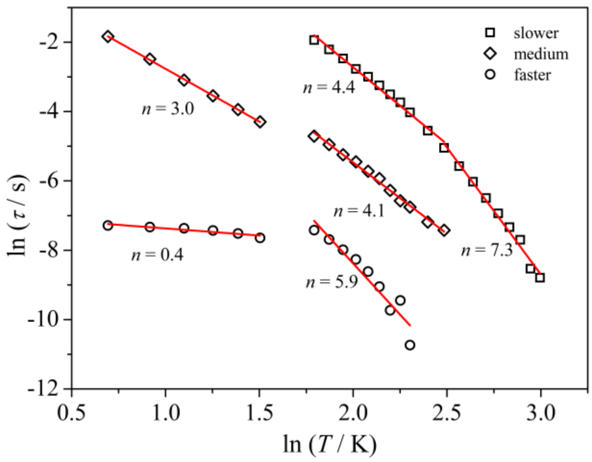

Figure S2. Plots of ln(T) *vs*. ln(τ). The solid red lines represent the best linear fit.

2. Computational details

The Dy_3 trinuclear complex has two types of molecular structures indicated as ***a*** and ***b***, respectively (Figure S3). Each of them has three types of magnetic center Dy^{3+} ions indicated as **Dy1**, **Dy2** and **Dy3**, respectively. Complete-active-space self-consistent field (CASSCF) calculations on individual Dy^{3+} fragments on the basis of single-crystal X-ray determined geometries at 30 K have been carried out with OpenMolcas program package.[S1] Each individual Dy^{3+} fragment was calculated keeping the experimentally determined structure of the corresponding compound while replacing the neighboring Dy^{3+} ion by diamagnetic Lu^{3+}.

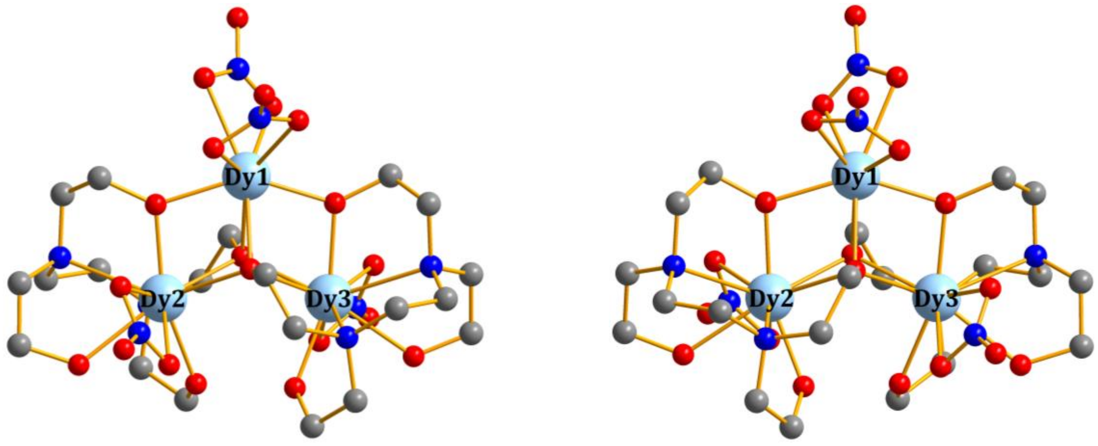

a ***b***

Figure S3. Calculated complete structures. H atoms are omitted for clarify.

The basis sets for all atoms are atomic natural orbitals from the OpenMolcas ANO-RCC library: ANO-RCC-VTZP for Dy^{3+}; VTZ for close O and N; VDZ for distant atoms. The calculations employed the second order Douglas-Kroll-Hess Hamiltonian, where scalar relativistic contractions were taken into account in the basis set and the spin-orbit couplings were handled separately in the restricted active space state interaction (RASSI-SO) procedure. Active electrons in 7 active orbitals include all *f* electrons (CAS (9 in 7) in the CASSCF calculation. To exclude all the doubts, we calculated all the roots in the active space. We have mixed the maximum number of spin-free state which was possible with our hardware (all from 21 sextets, 128 from 224 quadruplets, 130 from 490 doublets for Dy^{3+}). SINGLE_ANISO program was used to obtain the energy levels, ***g*** tensors, magnetic easy axes, *et al.* based on the above CASSCF/RASSI-SO calculations.[S2-S4]

The energy levels, *g* tensors and the predominant m_J values of the lowest eight Kramers doublets (KDs) of individual Dy^{3+} fragments are shown in Table S4. The m_J components for

the lowest eight KDs of individual Dy^{3+} fragments are shown in Table S5, where the ground KDs for individual Dy^{3+} fragments are all mostly composed by $m_J = \pm 15/2$. The first excited KDs of individual Dy^{3+} fragments are mostly composed by $m_J = \pm 13/2$. The second excited KDs of individual Dy^{3+} fragments are all composed by several m_J states severely, which leads to the large transversal magnetic moments in the second excited KDs for most of them.[S5,S6]

The above calculated magnetic properties such as energy barriers, ***g*** tensors, the predominant m_J values, *et al*. are only for individual Dy^{3+} fragments. However, although their magnetic anisotropies mainly come from individual Dy^{3+} ions, the Dy^{3+}-Dy^{3+} interactions have some certain influence on the slow magnetic relaxation process. The calculated ground g_z value of individual Dy^{3+} fragments are all close to 20, which shows that the Dy^{3+}-Dy^{3+} exchange interactions can be approximately regarded as the *Ising* type. POLY_ANISO program was used to fit the exchange coupling constants of J_{exch} and the intermolecular interactions zJ' through comparison of the computed and measured magnetic susceptibilities (Figures S4).

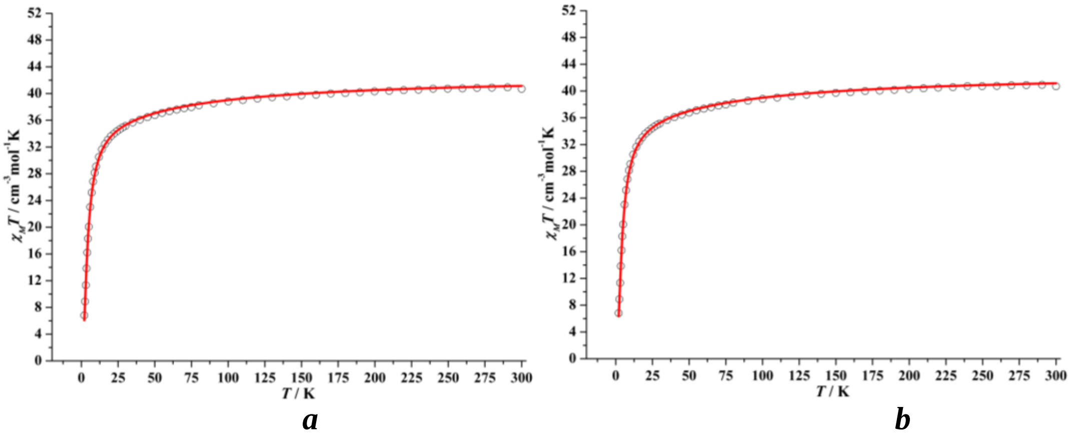

Figure S4. Calculated (red solid line) and experimental (black square dot) data of magnetic susceptibilities.

The parameters from Table S6 were calculated with respect to the pseudospin $\tilde{S}_{Dy} = 1/2$ of the Dy^{3+} ion. The dipolar magnetic coupling constants $\tilde{J}_{dip}$ were calculated exactly. The calculated and experimental $\chi_M T$ versus T plot of this SMM are shown in Figure S4, where the fits are all close to the experiments in the whole temperature range. From Table S6, the Dy^{3+}-Dy^{3+} interactions within Lines model are all antiferromagnetic. We gave the exchange energies, the transversal magnetic moments between each exchange doublets Δ_t and the main values of the g_z for the lowest four exchange doublets in Table S7, where the g_z value of the ground exchange states are 10.814 and 11.501, which confirm that the Dy^{3+}-Dy^{3+} interactions are all antiferromagnetic. The main magnetic axes on Dy^{3+} ions are indicated in Figures 1b, and the local magnetization vectors of Dy^{3+} ions can probably be approximately regarded as a triangle.

The included angles between main magnetic axes of Dy^{3+} ions are all larger than 97°. A vanishing small susceptibility at low temperature is ascribed to their close toroidal magnetic moments as well.

To fit the exchange interactions in the Dy_3 clusters, we took two steps to obtain it. Firstly, we calculated individual Dy^{3+} fragments using CASSCF/RASSI-SO to obtain the corresponding magnetic properties. Then, the exchange interaction between the magnetic centers was considered within the Lines model,[S7] while the account of the dipole-dipole magnetic coupling was treated exactly. The Lines model is effective and has been successfully used widely in the research field of *d* and *f*-elements single-molecule magnets.[S8,S9]

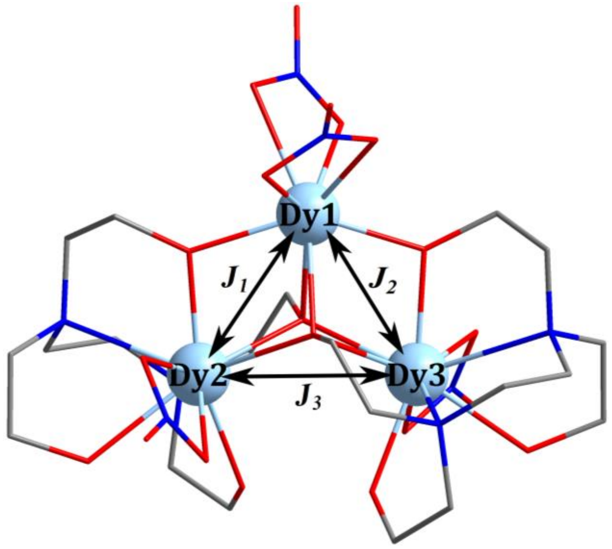

Figure S5. Scheme of the Dy^{3+}-Dy^{3+} interactions in the Dy_3 triangle clusters.

In this triangular Dy_3 SMM, there are three types of $\tilde{J}$ (Figure S5). The Ising exchange Hamiltonian is: $\hat{H}_{exch} = -\tilde{J}_1\hat{\tilde{S}}_{Dy1}\hat{\tilde{S}}_{Dy2} - \tilde{J}_2\hat{\tilde{S}}_{Dy1}\hat{\tilde{S}}_{Dy3} - \tilde{J}_3\hat{\tilde{S}}_{Dy2}\hat{\tilde{S}}_{Dy3}$ (S4), $\tilde{J} = 25\cos\varphi J$, where φ is the angle between the anisotropy axes on two Dy^{3+} sites, and J is the Lines exchange coupling parameter. $\tilde{S}_{Dy}$ = 1/2 is the ground pseudospin on the Dy^{3+} site. $\tilde{J}_{total}$ is the parameter of the total magnetic interaction ($\tilde{J}_{total} = \tilde{J}_{dip} + \tilde{J}_{exch}$) between magnetic center ions. The dipolar magnetic coupling can be calculated exactly, while the exchange coupling constant was fitted through comparison of the computed and measured magnetic susceptibilities using POLY_ANISO program.

3. Second-harmonic generation (SHG) characterization

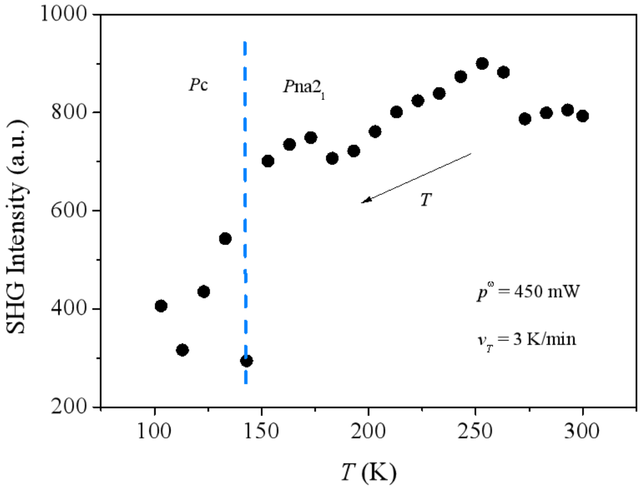

Figure S6. SHG intensity as a function of temperature in the temperature range of 100 to 300 K. The minimum around 150 K indicates a ferroelectric-ferroelectric transition due to the space group change from *Pc* to *P*na2_1.

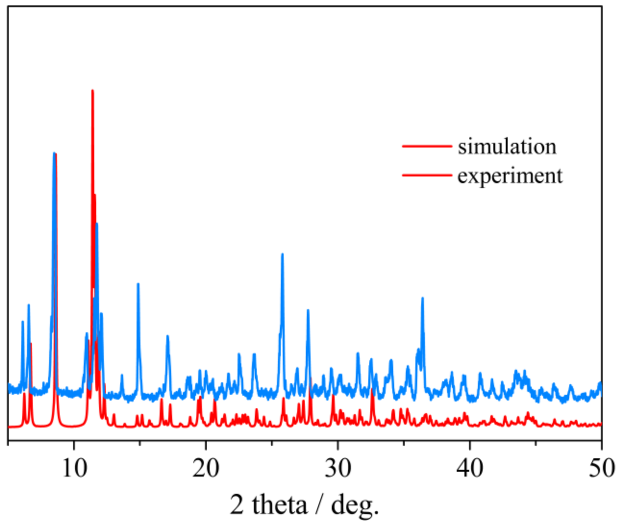

Figure S7. Powder-XRD patterns of **1**.

4. Tables

Table S1. Summary of space groups and cell parameters at 298 K and 30 K, respectively.

	**1**	
T (K)	298	30
Space group	*P*na2_1	*P*c
a (Å)	28.3864(6)	8.9395(3)
b (Å)	14.9148(3)	14.8628(6)
c (Å)	9.0995(2)	28.0678(12)
a/*b*	1.9032	0.6015
b/*c*	1.6390	0.5295
c/*a*	0.6101	3.1398
α (deg.)	90	90
β (deg.)	90	90.05
γ (deg.)	90	90
v(Å^3)	3852.52(14)	3729.3
Z	4	2
*D*c (g·cm^{-3})	2.154	2.225
μ (mm^{-1})	5.848	6.041
R_{int}	0.0238	0.0602
GOOF	1.060	1.277
R_1	0.0245	0.0914
wR_2	0.0477	0.2402
□ρ_{max} (e Å^{-3})	0.45	3.54
□ρ_{min} (e Å^{-3})	-0.57	-5.12
Flack	-0.024(12)	0.20(5)

Table S2. Continuous shape measure calculations for the Dy^{3+} ions at 298 K and 30 K.

T = 298 K						
Dy^{3+}	Dy1 [ML_8]	Dy2 [ML_9]	Dy3 [ML_9]			
Structure	D_{4d}	C_{4v}	C_{4v}			
Deviation value	3.501	2.367	2.456			
T = 30 K						
Dy^{3+}	Dy1A [ML_8]	Dy2A [ML_9]	Dy3A [ML_9]	Dy1B [ML_8]	Dy2B [ML_9]	Dy3B [ML_9]
Structure	D_{4d}	C_{4v}	C_{4v}	D_{4d}	C_{4v}	C_{4v}
Deviation value	3.523	2.518	2.401	3.390	2.989	2.475

D_{4d} = Square antiprism (SAPR-8);
C_{4v} = Capped square antiprism J10 (JCSAPR-9).

Table S3. The best fitting results of the temperature dependent relaxation times.

$\tau^{-1} = \mathrm{C} + \mathrm{B}T^{n} + \tau_0^{-1}\exp(-U_{\mathrm{eff}}/k_{\mathrm{B}}T)$			
	faster	medium	slower
C	1687	---	---
B	---	1.2	0.003
n	---	2.6	4.2
τ_0	1.0×10^{-8}	2.0×10^{-6}	7.0×10^{-8}
$U_{\mathrm{eff}}(\mathrm{cm}^{-1})$	55.6	50.04	110.0
dominated relaxation processes	QTM	Raman + Orbach	Raman + Orbach

Table S4. Calculated energy levels (cm^{-1}), ***g*** (g_x, g_y, g_z) tensors and predominant m_J values of the lowest eight Kramers doublets (KDs) of individual Dy^{3+} fragments using CASSCF/RASSI-SO with OpenMolcas.

KDs	***a*(Dy1)**			***a*(Dy2)**			***a*(Dy3)**		
	E/cm^{-1}	***g***	m_J	E/cm^{-1}	***g***	m_J	E/cm^{-1}	***g***	m_J
1	0.0	0.003<br>0.004<br>19.842	±15/2	0.0	0.014<br>0.018<br>19.799	±15/2	0.0	0.002<br>0.003<br>19.843	±15/2
2	234.0	0.057<br>0.075<br>17.027	±13/2	143.4	0.212<br>0.290<br>17.009	±13/2	236.3	0.065<br>0.073<br>17.091	±13/2
3	450.0	0.764<br>1.251<br>13.626	±11/2	316.8	1.874<br>5.967<br>10.581	±11/2	507.0	0.258<br>0.455<br>13.834	±11/2
4	505.1	0.107<br>0.478<br>18.863	±1/2	368.9	9.590<br>5.634<br>0.348	±1/2	680.8	2.903<br>4.410<br>8.668	±9/2
5	574.0	5.433<br>6.679<br>9.560	±9/2	421.1	1.392<br>5.047<br>10.233	±3/2	720.5	11.602<br>6.771<br>0.126	±1/2
6	675.3	0.421<br>1.178	±5/2	525.2	1.696<br>2.967	±5/2	764.3	5.675<br>3.171<br>0.296	±3/2

		14.308			12.612				
7	741.0	0.257<br>0.728<br>17.147	±1/2	550.5	0.299<br>1.540<br>16.511	±7/2	796.1	8.261<br>5.857<br>2.214	±5/2
8	1022.1	0.021<br>0.032<br>19.643	±5/2	622.5	0.692<br>1.535<br>16.303	±7/2	879.5	1.074<br>2.605<br>16.216	±7/2

KDs	***b*(Dy1)**			***b*(Dy2)**			***b*(Dy3)**		
	E/cm^{-1}	***g***	m_J	E/cm^{-1}	***g***	m_J	E/cm^{-1}	***g***	m_J
1	0.0	0.003<br>0.003<br>19.821	±15/2	0.0	0.004<br>0.004<br>19.833	±15/2	0.0	0.004<br>0.004<br>19.835	±15/2
2	227.2	0.089<br>0.109<br>17.010	±13/2	152.5	0.207<br>0.248<br>17.128	±13/2	241.4	0.062<br>0.075<br>17.044	±13/2
3	437.1	0.772<br>1.124<br>13.332	±11/2	326.2	2.844<br>4.400<br>9.545	±11/2	512.2	0.675<br>1.059<br>13.553	±11/2
4	496.2	0.543<br>1.897<br>17.606	±1/2	374.7	0.592<br>4.052<br>10.593	±1/2	653.5	2.248<br>3.849<br>14.521	±1/2
5	564.1	3.991<br>6.415<br>9.962	±9/2	416.8	0.039<br>3.551<br>8.012	±1/2	718.6	2.185<br>4.286<br>12.871	±1/2
6	619.3	1.080<br>1.552<br>16.602	±3/2	479.6	9.441<br>5.698<br>1.945	±9/2	792.7	1.388<br>2.752<br>10.877	±9/2
7	684.1	0.288<br>0.585	±7/2	552.2	1.637<br>2.831	±5/2	843.3	0.124<br>2.121	±5/2

		16.742			11.368			16.258	
8	953.6	0.030<br><br>0.032<br>19.594	±5/2	629.6	0.959<br><br>2.603<br>16.259	±7/2	906.8	0.955<br><br>2.049<br>16.684	±7/2

Table S5. Wave functions with definite projection of the total moment | m_J > for the lowest eight KDs of individual Dy^{3+} fragments.

<table>
<tr><th></th><th>E/cm^{-1}</th><th>wave functions</th></tr>
<tr><td rowspan="8">a(Dy1)</td><td>0.0</td><td>99.7%|±15/2></td></tr>
<tr><td>234.0</td><td>98.7%|±13/2></td></tr>
<tr><td>450.0</td><td>89.7%|±11/2>+2.6%|±9/2></td></tr>
<tr><td>505.1</td><td>39.2%|±1/2>+28.1%|±3/2>+15.9%|±5/2>+9.2%|±7/2></td></tr>
<tr><td>574.0</td><td>52.0%|±9/2>+17.8%|±7/2>+13.7%|±5/2>+9.0%|±3/2></td></tr>
<tr><td>675.3</td><td>24.5%|±5/2>+24.4%|±7/2>+23.3%|±9/2>+18.3%|±3/2></td></tr>
<tr><td>741.0</td><td>28.7%|±1/2>+26.2%|±7/2>+18.7%|±3/2>+16.9%|±5/2></td></tr>
<tr><td>1022.1</td><td>26.5%|±5/2>+24.0%|±3/2>+20.0%|±1/2>+19.8%|±7/2></td></tr>
<tr><td rowspan="8">a(Dy2)</td><td>0.0</td><td>99.1%|±15/2></td></tr>
<tr><td>143.4</td><td>92.4%|±13/2></td></tr>
<tr><td>316.8</td><td>41.3%|±11/2>+25.8%|±1/2>+13.3%|±3/2>+7.6%|±5/2></td></tr>
<tr><td>368.9</td><td>45.5%|±1/2>+28.4%|±11/2>+8.8%|±3/2>+7.3%|±5/2></td></tr>
<tr><td>421.1</td><td>49.9%|±3/2>+11.8%|±9/2>+11.3%|±1/2>+9.8%|±7/2>+8.3%|±5/2>+8.0%|±11/2></td></tr>
<tr><td>525.2</td><td>36.5%|±5/2>+18.6%|±9/2>+13.6%|±3/2>+12.7%|±1/2>+12.2%|±11/2></td></tr>
<tr><td>550.5</td><td>32.2%|±7/2>+26.9%|±5/2>+25.2%|±9/2>+10.2%|±3/2></td></tr>
<tr><td>622.5</td><td>47.5%|±7/2>+30.2%|±9/2>+13.2%|±5/2></td></tr>
<tr><td rowspan="8">a(Dy3)</td><td>0.0</td><td>99.7%|±15/2></td></tr>
<tr><td>236.3</td><td>97.5%|±13/2></td></tr>
<tr><td>507.0</td><td>87.9%|±11/2>+6.1%|±9/2></td></tr>
<tr><td>680.8</td><td>36.9%|±9/2>+28.2%|±1/2>+16.8%|±3/2>+8.3%|±7/2></td></tr>
<tr><td>720.5</td><td>59.7%|±1/2>+22.7%|±3/2>+5.9%|±5/2>+4.6%|±9/2></td></tr>
<tr><td>764.3</td><td>35.8%|±3/2>+28.9%|±9/2>+21.6%|±7/2>+9.5%|±1/2></td></tr>
<tr><td>796.1</td><td>58.0%|±5/2>+22.1%|±3/2>+10.8%|±7/2>+7.1%|±9/2></td></tr>
<tr><td>879.5</td><td>54.1%|±7/2>+25.4%|±5/2>+16.1%|±9/2></td></tr>
<tr><td rowspan="8">b(Dy1)</td><td>0.0</td><td>99.6%|±15/2></td></tr>
<tr><td>227.2</td><td>98.0%|±13/2></td></tr>
<tr><td>437.1</td><td>86.6%|±11/2>+4.2%|±9/2></td></tr>
<tr><td>496.2</td><td>40.6%|±1/2>+24.8%|±3/2>+14.0%|±5/2>+9.0%|±7/2>+8.1%|±9/2></td></tr>
<tr><td>564.1</td><td>44.7%|±9/2>+19.3%|±7/2>+15.3%|±5/2>+11.0%|±3/2></td></tr>
<tr><td>619.3</td><td>27.9%|±3/2>+23.0%|±5/2>+18.8%|±1/2>+15.8%|±9/2>+12.5%|±7/2></td></tr>
<tr><td>684.1</td><td>34.9%|±7/2>+21.3%|±5/2>+15.9%|±9/2>+13.2%|±1/2>+12.3%|±3/2></td></tr>
<tr><td>953.6</td><td>24.9%|±5/2>+22.2%|±7/2>+21.0%|±3/2>+18.0%|±1/2>+10.8%|±9/2></td></tr>
<tr><td rowspan="5">b(Dy2)</td><td>0.0</td><td>99.3%|±15/2></td></tr>
<tr><td>152.5</td><td>91.5%|±13/2></td></tr>
<tr><td rowspan="2">326.2</td><td>45.3%|±11/2>+18.2%|±1/2>+15.5%|±3/2>+8.8%|±5/2>+8.7%|±9/2></td></tr>
<tr><td></td></tr>
<tr><td></td><td></td></tr>
</table>

<table>
<tr><td rowspan="5"></td><td>374.7</td><td>37.4%|±1/2>+23.4%|±3/2>+17.9%|±11/2>+10.1%|±9/2>+8.0%|±5/2></td></tr>
<tr><td>416.8</td><td>28.2%|±1/2>+19.1%|±5/2>+15.4%|±7/2>+14.6%|±3/2>+14.5%|±11/2></td></tr>
<tr><td>479.6</td><td>27.7%|±9/2>+23.8%|±7/2>+21.2%|±3/2>+12.2%|±5/2>+7.5%|±1/2></td></tr>
<tr><td>552.2</td><td>26.3%|±5/2>+26.1%|±9/2>+20.7%|±3/2>+13.1%|±7/2>+7.9%|±1/2></td></tr>
<tr><td>629.6</td><td>43.4%|±7/2>+25.5%|±5/2>+22.2%|±9/2></td></tr>
<tr><td rowspan="8">b(Dy3)</td><td>0.0</td><td>99.6%|±15/2></td></tr>
<tr><td>241.4</td><td>97.6%|±13/2></td></tr>
<tr><td>512.2</td><td>87.5%|±11/2>+4.2%|±9/2></td></tr>
<tr><td>653.5</td><td>28.7%|±1/2>+25.6%|±3/2>+18.1%|±9/2>+14.7%|±5/2>+8.1%|±7/2></td></tr>
<tr><td>718.6</td><td>40.9%|±1/2>+31.8%|±9/2>+16.2%|±3/2>+6.2%|±7/2></td></tr>
<tr><td>792.7</td><td>29.8%|±9/2>+22.5%|±3/2>+19.3%|±7/2>+12.3%|±5/2>+11.9%|±1/2></td></tr>
<tr><td>843.3</td><td>36.2%|±5/2>+27.6%|±3/2>+18.1%|±7/2>+13.6%|±1/2></td></tr>
<tr><td>906.8</td><td>45.9%|±7/2>+30.7%|±5/2>+12.2%|±9/2>+7.2%|±3/2></td></tr>
</table>

Table S6. Fitted exchange couplings $\tilde{J}_{exch}$, the calculated dipole-dipole interactions $\tilde{J}_{dip}$ and the total constants $\tilde{J}_{total}$ between magnetic center ions in this cluster (cm^{-1}). The intermolecular interactions *zJ′* of ***a*** and ***b*** were both fitted to –0.03 cm^{-1}.

	a			***b***		
	J_1	J_2	J_3	J_1	J_2	J_3
$\tilde{J}_{dip}$	–5.3	–5.3	–3.0	–5.5	–5.5	–3.0
$\tilde{J}_{exch}$	–0.4	–0.1	–2.2	0.0	–0.6	–2.3
$\tilde{J}_{total}$	–5.7	–5.4	–5.2	–5.5	–6.1	–5.3

Table S7. Exchange energies *E* (cm^{-1}), the transversal magnetic moments between each exchange doublets Δ_t (μ_B) and the main values of the g_z for the lowest four exchange doublets.

	a			***b***		
	E	Δ_t	g_z	*E*	Δ_t	g_z
1	0.000	0.285×10^{-10}	10.814	0.000	0.109×10^{-10}	11.501
2	5.354	0.220×10^{-4}	44.245	5.329	0.935×10^{-8}	42.343
3	5.361	0.336×10^{-4}	42.317	5.561	0.971×10^{-8}	44.685
4	5.618	0.766×10^{-8}	29.163	5.743	0.228×10^{-8}	28.222

References

[S1] F. Aquilante, J. Autschbach, R. K. Carlson, L. F. Chibotaru, M. G. Delcey, L. De Vico, I. F. Galván, N. Ferré, L. M. Frutos, L. Gagliardi, M. Garavelli, A. Giussani, C. E. Hoyer, G. Li Manni, H. Lischka, D. Ma, P. Å. Malmqvist, T. Müller, A. Nenov, M. Olivucci, T. B. Pedersen, D. Peng, F. Plasser, B. Pritchard, M. Reiher, I. Rivalta, I. Schapiro, J. Segarra-Martí, M. Stenrup, D. G. Truhlar, L. Ungur, A. Valentini, S. Vancoillie, V. Veryazov, V. P. Vysotskiy, O. Weingart, F. Zapata, R. Lindh, *J. Comput. Chem*. **2016**, *37*, 506−541.

[S2] L. F. Chibotaru, L. Ungur, A. Soncini, *Angew. Chem., Int. Ed*. **2018**, *47*, 4126–4129.

[S3] L. Ungur, W. Van den Heuvel, L. F. Chibotaru, *New J. Chem.* **2009**, *33*, 1224–1230.

[S4] L. F. Chibotaru, L. Ungur, C. Aronica, H. Elmoll, G. Pilet, D. Luneau, *J. Am. Chem. Soc*. **2008**, *130*, 12445–12455).

[S5] A. Lunghi, F. Totti, R. Sessoli, S. Sanvito, *Nat. Commun.* **2017**, *8*, 14620–14626.

[S6] F. Lu, M. M. Ding, J. X. Li, B. L. Wang, Y. Q. Zhang, *Dalton Trans*. **2020**, *49*, 14576–14583.

[S7] M. E. Lines, *J. Chem. Phys*. **1971**, ***55***, 2977–2984.

[S8] K. C. Mondal, A. Sundt, Y. H. Lan, G. E. Kostakis, O. Waldmann, L. Ungur, L. F. Chibotaru, C. E. Anson, A. K. Powell, *Angew. Chem., Int. Ed.* **2012**, *51*, 7550–7554.

[S9] S. K. Langley, D. P. Wielechowski, V. Vieru, N. F. Chilton, B. Moubaraki, B. F. Abrahams, L. F. Chibotaru, K. S. Murray, *Angew. Chem., Int. Ed*. **2013**, *52*, 12014–12019.
